# Microfluidic-Based Biosensor for Blood Viscosity and Erythrocyte Sedimentation Rate Using Disposable Fluid Delivery System

**DOI:** 10.3390/mi11020215

**Published:** 2020-02-20

**Authors:** Yang Jun Kang

**Affiliations:** Department of Mechanical Engineering, Chosun University, 309 Pilmun-daero, Dong-gu, Gwangju 61452, Korea; yjkang2011@chosun.ac.kr; Tel.: +82-62-230-7052; Fax: +82-62-230-7055

**Keywords:** blood viscosity, Erythrocyte sedimentation rate (ESR), T-shaped microfluidic channel, air-compressed syringe (ACS), micro-particle image velocimetry

## Abstract

To quantify the variation of red blood cells (RBCs) or plasma proteins in blood samples effectively, it is necessary to measure blood viscosity and erythrocyte sedimentation rate (ESR) simultaneously. Conventional microfluidic measurement methods require two syringe pumps to control flow rates of both fluids. In this study, instead of two syringe pumps, two air-compressed syringes (ACSs) are newly adopted for delivering blood samples and reference fluid into a T-shaped microfluidic channel. Under fluid delivery with two ACS, the flow rate of each fluid is not specified over time. To obtain velocity fields of reference fluid consistently, RBCs suspended in 40% glycerin solution (hematocrit = 7%) as the reference fluid is newly selected for avoiding RBCs sedimentation in ACS. A calibration curve is obtained by evaluating the relationship between averaged velocity obtained with micro-particle image velocimetry (μPIV) and flow rate of a syringe pump with respect to blood samples and reference fluid. By installing the ACSs horizontally, ESR is obtained by monitoring the image intensity of the blood sample. The averaged velocities of the blood sample and reference fluid (<*U_B_*>, <*U_R_*>) and the interfacial location in both fluids (*α_B_*) are obtained with μPIV and digital image processing, respectively. Blood viscosity is then measured by using a parallel co-flowing method with a correction factor. The ESR is quantified as two indices (*t_ESR_*, *I_ESR_*) from image intensity of blood sample (<*I_B_*>) over time. As a demonstration, the proposed method is employed to quantify contributions of hematocrit (*Hct* = 30%, 40%, and 50%), base solution (1× phosphate-buffered saline [PBS], plasma, and dextran solution), and hardened RBCs to blood viscosity and ESR, respectively. Experimental Results of the present method were comparable with those of the previous method. In conclusion, the proposed method has the ability to measure blood viscosity and ESR consistently, under fluid delivery of two ACSs.

## 1. Introduction

Microcirculation plays a substantial role in regulating blood flows and exchanging substances (gases, nutrients, and waste) between blood samples and peripheral tissues. Impaired microcirculation commonly leads to organ failures or mortality [1]. There is a need for comprehensive research that offers an insight that intrinsic properties and flow characteristics of blood samples share with microcirculatory disorders such as hypertension, sickle cell anemia, and diabetes [2]. The previous study has reported that biophysical properties of blood samples (hematocrit (*Hct*), viscosity, and erythrocyte sedimentation rate (ESR)) are strongly correlated with coronary heart diseases [3]. Thereafter, the biophysical properties of the blood sample have been studied extensively for the effective monitoring of circulatory disorders [4,5,6,7,8,9].

Under normal physiological conditions, red blood cells (RBCs) occupy 40–50% of blood volume. As RBCs are the most abundant cells in the blood sample, the biophysical properties of the blood sample are determined dominantly by properties of RBCs. The characteristics of RBCs, including morphology, membrane viscoelasticity, and RBCs count, are evaluated by quantifying several biophysical properties of blood samples, including viscoelasticity (or viscosity), deformability, and hematocrit. In that regard, plasma proteins in blood samples induce RBC aggregation, which occurs at an extremely low shear rate (i.e., $\dot{\gamma }$ = 1~10 s^−1^) [10] or stasis. Among the biophysical properties of blood samples, blood viscosity is determined by several factors, including hematocrit, plasma viscosity, RBCs deformability, and RBCs aggregation. Thus, their properties of blood samples are employed to monitor variations in the characteristics of blood samples. At lower shear rates, RBC aggregation causes to increase blood viscosity. At high shear rates, the deformation and alignment of RBCs lead to a decrease in blood viscosity. In other words, blood viscosity provides information on aggregation and deformability simultaneously. However, at extremely low shear rates, a syringe pump (SP) exhibits fluidic instability and RBC sedimentation continuously occurs. A microfluidics-based viscometer does not provide consistent values of blood viscosity. Conventionally, blood viscosity has been measured at sufficiently high shear rates (i.e., $\dot{\gamma }$ > 10 s^−1^ [11,12] or $\dot{\gamma }$ > 50–100 s^−1^ [13,14]), especially in microfluidic environments. For the reason, blood viscosity obtained with a microfluidic device does not give sufficient information on the contributions of plasma proteins to RBC aggregation. To evaluate variations in plasma proteins consistently, it is additionally necessary to quantify RBCs aggregation or ESR.

A microfluidic device has several advantages, including small volume consumption, fast measurement, easy sample handling, high sensitivity, and disposability. Thus, it has been widely used to measure various biophysical properties of blood samples (i.e., blood viscosity [15], RBCs aggregation [16], RBCs deformability [17,18], and hematocrit [19]).

The previous methods for measuring blood viscosity are conveniently divided into three categories (i.e., driving sources, devices, and quantification techniques). First, extrinsic driving sources such as SPs [20], pressure sources, and hand-held pipettes [13] have been suggested for delivering a blood sample into a specific device. Additionally, intrinsic driving sources such as capillary force (or surface tension) [21,22] and gravity force [23] have been applied to supply blood samples into a device. Second, various devices such as a microelectromechanical system (MEMS)-based microfluidic device, a 3D-printed microfluidic device [13,24], and a paper-based device [25] have been suggested for inducing blood flow in a specifically constrained direction. Third, quantification techniques such as advancing meniscus (i.e., variations of a blood column over time) [15,22,26,27], the falling time of a metal sphere in a tube [28], electric impedances (i.e., resistance, capacitance) [29,30], droplet length [31], digital flow compartment with a microfluidic channel array [11,12], interface detection in co-flowing streams [32,33], and reversal flow switching in a Wheatstone bridge analog of a fluidic circuit [14] have been suggested to measure blood viscosity.

To measure RBCs aggregation in microfluidic environments, a blood sample is placed into a microfluidic channel. By applying shear stress to the blood sample with external driving systems (i.e., an SP [34], pinch valve [16], or stirring motor [35]), the RBCs in the blood sample are aggregated or disaggregated, depending on the shear rate. Several quantification methods, such as light intensity (i.e., transmission, and back-scattering) [16], electrical conductivity [36,37], microscopic RBC images [38,39,40], ultrasonic images [41], and optical tweezers [42] have been suggested for obtaining temporal variations of RBCs aggregation. As another approach, RBC aggregation can be quantified by measuring the sedimentation distances of RBCs in a blood sample during a specific duration (i.e., ESR). Unlike the conventional Westergren ESR method, a microfluidic-based ESR measurement is quantified by measuring the conductivity of the blood sample in a PDMS chamber with a square cross-section (i.e., each side = 4 mm, depth = 5 mm) [43]. Owing to the continuous ESR in the driving syringe, RBC-free regions (or depleted regions) expand from the top layer with an elapse of time. The blood sample is supplied into a microfluidic device from the top layer of the driving syringe. To monitor blood flows in the microfluidic channel, microscopic images are sequentially captured with a high speed camera. Image intensity of each microscopic image is calculated over time by conducting digital image processing. The ESR is then evaluated by quantifying temporal variations of the image intensity [44].

To measure blood viscosity and RBC aggregation inexpensively, two SPs should be replaced with an inexpensive and disposable delivery system. To remove the syringe pump, single ACS is suggested to infuse the blood sample into a microfluidic device for measuring pressure and RBCs aggregation over continuously varying flow rates [45]. In this study, the ultimate goal of this study is to measure blood viscosity and RBC aggregation (or ESR), without two SPs.

In this study, a simple method for measuring blood viscosity and ESR is proposed. It involves the quantification of the interfacial location in a co-flowing channel and microscopic image intensity of blood sample flowing in a microfluidic device. Two air-compressed syringes (ACSs) are employed to simultaneously deliver the blood sample and reference fluid. Based on an ACS for delivering blood samples as suggested in previous studies [46,47], two ACSs are suggested to deliver blood samples and reference fluid simultaneously. Since the flow rates of both fluids are not specified under fluid delivery with the ACSs, it is necessary to quantify them with a time-resolved micro-particle image velocimetry (µ-PIV) technique. Based on a parallel co-flowing method with a correction factor [32], the blood viscosity is measured by monitoring the interfacial location in a co-flowing channel. Unlike the previous studies [46,47], two ACSs are installed horizontally to measure ESR effectively. Continuous sedimentation in the ACS causes an expansion of an RBC-free layer from the top layer. When blood samples are delivered to the blood channel from the ACS, the populations of RBCs (or hematocrit) are reduced over time. Since a continuous ESR contributes to increasing microscopic image intensity of blood flows, the ESR can be quantified by monitoring the image intensity of the blood sample.

When compared to previous methods that have the ability to measure blood viscosity under fluid delivery with syringe pumps, two syringe pumps are replaced by two ACSs as a novelty of this method. Here, a 40% glycerin solution is newly selected as the reference fluid. RBCs as fluid tracers are added into reference fluid. Velocity fields of both fluids are obtained consistently over time by conducting a time-resolved micro-PIV technique.

By installing the ACSs horizontally, the continuous ESR inside the ACS is filled with the blood sample causing it to expand RBC-free regions. As RBCs aggregation tends to increase substantially at lower hematocrit or lower velocity, it contributes to increasing the image intensity of blood samples. Thus, it is possible to evaluate the ESR by monitoring the microscopic image intensity of the blood sample.

## 2. Materials and Methods

### 2.1. Fabrication of Microfluidic Device and Experimental Procedure

**Figure 1:**
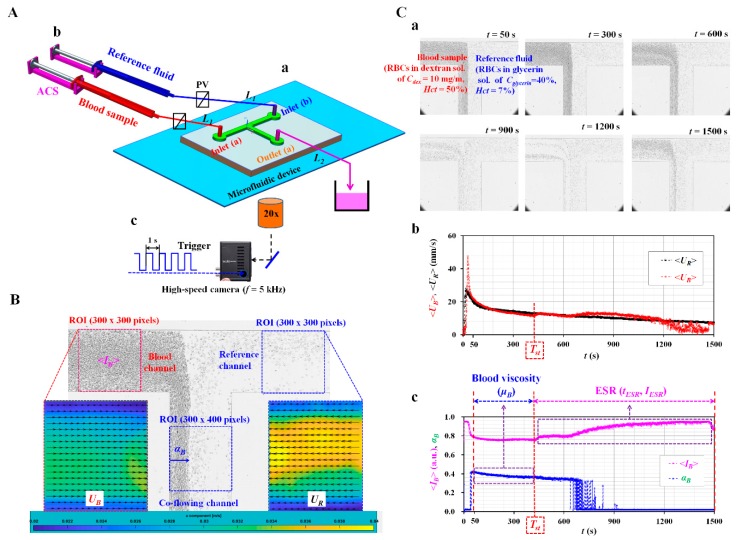
Proposed method for measuring blood viscosity and erythrocyte sedimentation rate (ESR) under fluid delivery of two air-compressed syringes (ACSs). (**A**) Schematic diagram of the proposed technique, including a microfluidic device, two ACSs, and an image acquisition system. (**a**) A microfluidic device consisting of two inlets (**a**,**b**), one outlet (**a**), and a T-shaped channel (i.e., blood channel, reference channel, and co-flowing channel). (**b**) Two ACSs for delivering blood samples and reference fluid. Each ACS was composed of a disposable syringe (~1 mL), a fixture, and a pinch valve. (**c**) The microfluidic device is located in an optical image acquisition system composed of optical microscopy with a 20× objective lens (NA = 0.4), and a high-speed camera. The camera had a frame rate of 5 kHz and captured sequential snapshots at an interval of 1 s. (**B**) Three regions-of-interest (ROIs) were selected for evaluating four parameters (<*I_B_*>, *U_B_*, *U_R_*, and *α_B_*). <*I_B_*> and *α_B_* were obtained by conducting digital image processing. *U_B_* and *U_R_* were obtained by conducting a micro-particle image velocimetry (PIV) technique. (**C**) As a preliminary demonstration, blood sample (normal RBCs in 10 mg/mL dextran solution (*Hct* = 50%)) and reference fluid (RBCs in 40% glycerin solution (*Hct* = 7%)) were delivered to each inlet with two ACSs. (**a**) Microscopic images captured at a specific time (*t*) (*t* = 50, 300, 600, 900, 1200, and 1500 s). (**b**) Temporal variations of <*U_B_*> and <*U_R_*>. (**c**) Temporal variations of <*I_B_*> and *α_B_*. Separation time (*T_st_*) was obtained as the time when <*I_B_*> started to increase. First, blood viscosity was evaluated from three parameters (*U_B_*, *U_R_*, and *α_B_*) obtained within *T_st_*. Second, the ESR of the blood sample was evaluated from <*I_B_*> obtained above *T_st_*.

A microfluidic device for measuring blood viscosity and ESR consisted of two inlets (a, b), one outlet (a), and a T-shaped channel (width = 250 μm, depth = 20 μm), as shown in Figure 1A-a. The T-shaped channel was composed of a blood channel, a reference channel, and a co-flowing channel. When analyzing the velocity fields of each fluid, the T-shaped channel does not require to align each microscopic image in the horizontal direction. Conventional micro-electromechanical-system techniques, such as photolithography and deep reactive ion etching (DRIE), were employed to fabricate 4-inch silicon mold. To peel off PDMS block from the master mold easily, plasma surface treatment was conducted after the DRIE process [48]. PDMS elastomer (Sylgard 184, Dow Corning, Midland, MI, USA) was mixed with a curing agent at a ratio of 10:1. After positioning the mold on a petri dish, the PDMS mixture was poured into the mold. Air bubbles dissolved in the PDMS were removed by operating a vacuum pump (WOB-L Pump, Welch, Gardner Denver, Milwaukee, WI, and USA) for 1 h. After curing the PDMS in a convective oven at 70 °C for 1 h, a PDMS block was peeled off from the mold. It cut with a razor blade. Two inlets and outlets were punched with a biopsy punch (outer diameter = 1.0 mm). After treating the surfaces of the PDMS block and a glass slide with an oxygen plasma system (CUTE-MPR, Femto Science Co., Gyeonggi-do, Korea), the PDMS block was bonded on a glass substrate. A microfluidic device was finally prepared by placing it on a hotplate at 120 °C for 10 min.

As shown in Figure 1A-b, two polyethylene tubes (*L*_1_) (length = 300 mm, inner diameter = 500 μm, and thickness = 500 μm) were tightly fitted into two inlets (a, b). The end of each tube was connected to the individual syringe needle of the ACS. The outlet of each ACS was clamped with a pinch valve. The other tube (*L*_2_) (length = 200 mm, inner diameter = 500 μm, and thickness = 500 μm) was tightly fitted into outlet (a). The end of the tube (*L*_2_) was connected to a waste dish. To remove air bubbles and avoid non-specific binding of plasma proteins to the inner surface of the channels, the channel was filled with bovine serum albumin (BSA) solution (*C_BSA_* = 2 mg/mL) through outlet (a). After an elapse of 10 min, the microfluidic channel was newly filled with 1× PBS.

**Figure A1:**
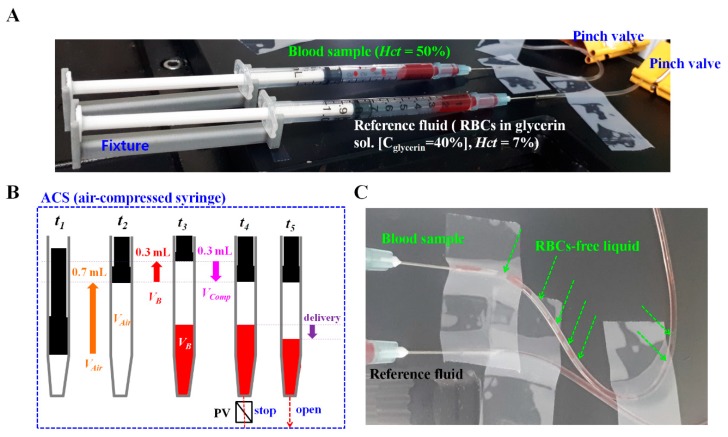
Fabrication and operation of the air-compressed syringe. (**A**) Two air-compressed syringes (ACSs) filled with blood sample (*Hct* = 50%) and reference fluid (RBCs in 40% glycerin sol. [*Hct* = 7%]). Each ACS was composed of a disposable syringe (~ 1mL), a fixture, and a pinch valve. (**B**) Operation of ACS: piston movement at the lowest position forward at *t* = *t_1_*, air suction (*V_Air_* = 0.7 mL) by moving piston to 0.7 mL backward at *t* = *t_2_*, blood suction by moving piston to 0.3 mL backward (*V_B_* = 0.3 mL) at *t* = *t_3_*, air compression by moving piston to 0.3 mL forward (*V_comp_* = 0.3 mL) at *t* = *t_4_*, and blood delivery by removing pinch valve at *t* = *t_5_*. Similarly, the reference fluid was sucked into the syringe instead of blood. The remaining procedure was the same as blood delivery with the ACS. The blood sample and reference fluid were then delivered into the corresponding inlets with two ACSs. (**C**) Snapshots for showing RBC-free liquid in a tube under blood delivery using an ACS. Owing to the continuous erythrocyte sedimentation rate (ESR) inside the ACS, a red blood cell (RBC)-free liquid was observed in the microfluidic channel after a certain lapse of time.

Based on the concept of ACS as reported in a previous study [45], two ACSs were employed to deliver the blood sample and reference fluid into the microfluidic device. Figure A1A (Appendix A) showed two ACSs filled with the blood sample (*Hct* = 50%) and reference fluid (RBCs suspended in 40% glycerin solution (*Hct* = 7%)). Each ACS was composed of a disposable syringe (~ 1 mL), a fixture, and a pinch valve. Two pinch valves were used to stop or allow the fluid flow of each fluid. Each ACS was placed horizontally on the stage of the optical microscope and fixed with an adhesive tape. Here, an angle of inclination of the ACS only depended on an individual fixture. It was certain that the installation angle of the ACS remained identical because the same fixture of the ACS was used for all experiments.

As shown in Figure A1B (Appendix A), the operation of each ACS was classified into five steps: (1) piston movement at the lowest position forward at *t* = *t*_1_, (2) air suction by moving the piston to 0.7 mL backward at *t* = *t*_2_, (3) blood suction by moving the piston to 0.3 mL backward at *t* = *t*_3_, (4) air compression by moving the piston to 0.3 mL forward at *t* = *t*_4_, and (5) blood delivery by removing the pinch valve at *t* = *t*_5_. As the air cavity inside the ACS was compressed to 0.3 mL, internal pressure increased substantially above atmospheric pressure. Similarly, the reference fluid was sucked into the syringe. The remainder of the procedure was the same as blood delivery. By removing two pinch valves, blood sample and reference fluid were delivered to the corresponding inlets because pressure difference increased inside the ACS.

The microfluidic device was positioned on an optical microscope (BX51, Olympus, Tokyo, Japan) equipped with a 20× objective lens (NA = 0.4). As shown in Figure 1A-c, a high-speed camera (FASTCAM MINI, Photron, Tokyo, Japan) was used to obtain sequential microscopic images of the blood sample and reference fluid flowing in the microfluidic channels. The camera offered a spatial resolution of 1280 × 1000 pixels. Each pixel corresponded to 10 μm physically. A function generator (WF1944B, NF Corporation, Yokohama, Japan) triggered the high-speed camera at an interval of 1 s. Then, two microscopic images were captured at a frame rate of 5 kHz.

To minimize the effect of temperature on blood viscosity, all experiments were conducted at a room temperature of 25 °C. Contributions of two factors (i.e., humidity, and atmospheric pressure) to the present method were neglected. After the blood sample was injected into an ACS, the blood sample did not contact with environment air. Additionally, the ACS was operated by pressure difference (Δ*P*) between pressure inside ACS (*P_ACS_*) and atmosphere pressure (*P_atm_*) (i.e., Δ*P* = *P_ACS_* − *P_atm_*) [47]. The pressure difference depended on air volume inside the ACS (i.e., gauge pressure), rather than atmospheric pressure.

### 2.2. Quantification of Microscopic Image Intensity, Blood Velocity Fields, and Interfacial Location

First, blood viscosity was obtained by quantifying the velocity fields of blood sample flowing in the blood channel, the velocity fields of reference fluid flowing in the reference channel, and the interface location between two fluids flowing in the co-flowing channel.

RBCs as fluid tracers were added into reference fluid to obtain the velocity fields of the reference fluid. To measure velocity fields of reference fluid consistently, RBCs should be distributed uniformly in reference fluid during experiments. According to previous studies [1,2], when reference fluid was prepared by adding RBCs into 1× PBS and filled into the ACS, sedimentation of RBCs in ACS occurred continuously over time. RBCs in reference fluid did not flow uniformly over time. After a certain lapse of time, there were no fluid tracers in reference fluid. It was then impossible to obtain the velocity fields of the reference fluid. To resolve the critical issue, a 40% glycerin solution was carefully selected as a base solution in reference fluid. Additionally, to minimize contributions of RBCs to velocity fields and viscosity, hematocrit of RBCs added into reference fluid was fixed at *Hct* = 7%.

As shown in Figure 1B, an ROI (300 × 300 pixels) was selected to obtain the velocity fields of the blood sample flowing in the blood channel. Another ROI with 300 x 300 pixels was selected to obtain the velocity fields of the reference fluid flowing in the reference channel. By conducting a time-resolved µPIV technique, the velocity fields of the blood sample (*U_B_*) across the blood channel width were obtained over time. Additionally, velocity fields of the reference fluid flow (*U_R_*) across the reference channel width were obtained over time. The size of the interrogation window was selected as 64 × 64 pixels. The window overlap was set to 75%. The velocity fields were validated and corrected with a median filter. The averaged velocities (<*U_B_*>, <*U_R_*>) of both fluids were calculated as an arithmetic average over the specific ROI. To obtain the interface (i.e., blood sample-filled width) in the co-flowing channel (*α_B_*), an ROI with 300 × 400 pixels was selected in the co-flowing channel. A gray-scale microscopic image was converted into a binary image by adopting Otsu’s method [49]. By conducting an arithmetic average over the ROI, variations of the interfacial location in the co-flowing channel (*α_B_*) were obtained over a period of time.

Second, the ESR was evaluated by quantifying the microscopic image intensity of the blood sample flowing in the blood channel. To evaluate the microscopic image intensity of blood flows, and ROI with 300 x 300 pixels was selected in the blood channel. The image intensity of the blood sample flowing in the blood channel was obtained by conducting digital image processing with a commercial software package (Matlab 2019, Mathworks, Natick, MA, USA). An averaged value of microscopic image intensity (<*I_B_*>) was obtained by performing an arithmetic average of image intensity over the specific ROI.

### 2.3. Quantification of Blood Viscosity and ESR

As a preliminary demonstration, blood samples (normal RBCs suspended in specific dextran solution (10 mg/mL), *Hct* = 50%) and reference fluid were delivered to the corresponding inlets (a, b) under the fluid delivery with two ACSs. To visualize the velocity fields of the reference fluid flowing in the reference channel, the reference fluid was prepared by adding normal RBCs (*Hct* = 7%) into a 40% glycerin solution.

**Figure A2:**
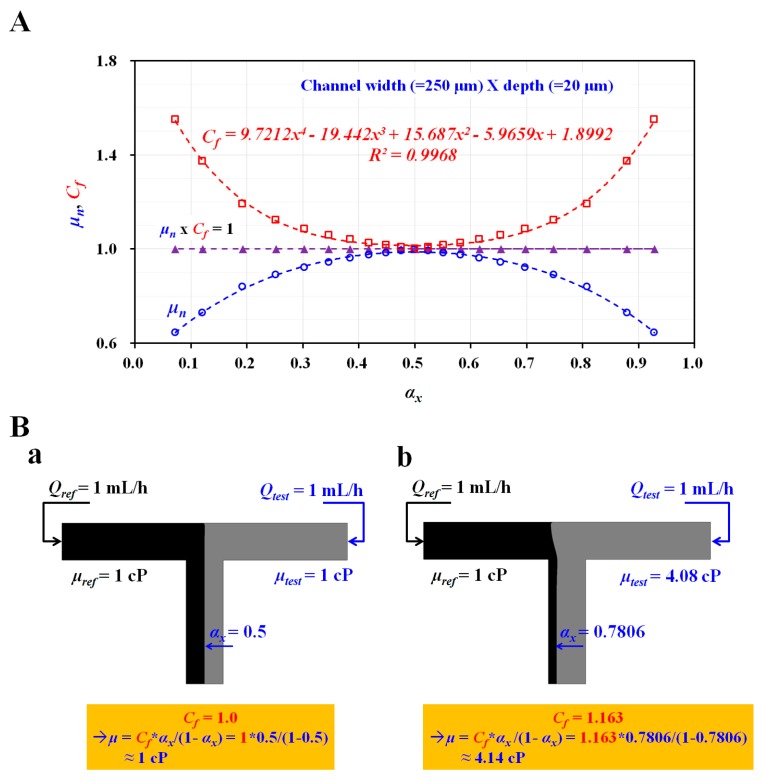
A correction factor of the parallel flow method estimated by conducting a numerical simulation. (**A**) The polynomial formula of a correction factor (*C_f_*) estimated from a numerical simulation. (**B**) Numerical simulation results for showing interfacial location depending on the viscosity of test fluid. (**a**) *α_x_* = 0.5 for *μ_test_* = 1 cP. Viscosity of test fluid was estimated as 1 cP by considering correction factor of *C_f_* = 1. (**b**) *α_x_* = 0.7806 for *μ_test_* = 4.08 cP. The viscosity of test fluid was estimated as 4.14 cP by considering the correction factor of *C_f_* = 1.163.

Figure 1C-a showed microscopic images captured at specific times (*t*) (*t* = 50, 300, 600, 900, 1200, and 1500 s). Above *t* = 600 s, the populations of RBCs flowing in the blood channel decreased substantially over time. As shown in Figure 1C-b, temporal variations of *U_B_* and *U_R_* were obtained by conducting the µPIV technique. As the pressure difference between the inner pressure and atmospheric pressure tended to decrease over time in the ACS, the averaged velocity of the reference fluid (<*U_R_*>) tended to decrease stably over time. However, owing to the continuous ESR inside the ACS, an RBC-free liquid was observed in a tube, as shown in Figure A1C (Appendix A). The averaged velocity of the blood sample (<*U_B_*>) varied unstably above *t* = 400 s. Figure 1C-c showed the temporal variations in the image intensity of the blood sample flowing in the blood channel (<*I_B_*>), and the interface between the two fluids in the co-flowing channel (*α_B_*). Similar to *U_B_*, the continuous ESR inside the ACS led to unstable behaviors in <*I_B_*> and *α_B_*. In this study, the separation time when unstable behavior began was denoted as *T_st_*. At *t* < *T_st_*, three factors (<*U_B_*>, <*U_R_*>, and <*α_B_*>) exhibited stable variations over time. Thus, the blood viscosity was quantified from the three factors (<*U_B_*>, <*U_R_*>, and *α_B_*). For a rectangular channel with an extremely low aspect ratio, an approximate formula of fluidic resistance was derived approximately as $R=\frac{12\;\mu_{B}\;L\;}{w\;h^{3}\;}$. A co-flowing channel was filled with a blood sample and reference fluid, respectively. For simple mathematical representation, both streams were represented as two fluidic resistances connected in parallel. The corresponding fluidic resistance for each fluid was derived as *R_B_* =$\;\frac{12\;\mu_{B}\;L}{W\alpha_{B}h^{3}}$ for a blood sample, and *R_R_* =$\;\frac{12\;\mu_{R}\;L}{W(1-\alpha_{B})\;h^{3}}$ for reference fluid. Here, *μ_R_* meant the viscosity of the reference fluid. As both fluids had the same pressure drop (i.e., Δ*P* = *R_B_*·*Q_B_* = *R_R_*·*Q_R_*), blood viscosity formula (*μ_B_*) was derived as $\mu_{B}=\mu_{R\;}\times \left(\frac{\alpha_{B}}{1-\alpha_{B}}\right)\times \left(\frac{Q_{R}}{Q_{B}}\right)$. Here, *Q_B_* and *Q_R_* represented the flow rate of the blood sample and reference fluid, respectively. The simple mathematical model did not account for real boundary conditions in co-flowing flows. Thus, to compensate for the deviation from the real boundary condition, the previous study included a correction factor in the analytical formula of blood viscosity. According to the blood viscosity formula reported in a parallel co-flowing method with a correction factor [32], the blood viscosity formula (*μ_B_*) was modified as $\mu_{B}=C_{f}\times \mu_{R\;}\times \left(\frac{\alpha_{B}}{1-\alpha_{B}}\right)\times \left(\frac{Q_{R}}{Q_{B}}\right)$. Since the correction factor (*C_f_*) was varied depending on the channel size, a numerical simulation was conducted to determine the correction factor. Based on a procedure discussed in a previous study [32], a numerical simulation using commercial computational fluid dynamics (CFD) software (CFD ACE+, ESI Group, Paris, France) for a rectangular channel (width = 250 μm, depth = 20 μm) was conducted to obtain the viscosity of the test fluid with respect to the interface. For convenience, it was assumed that the reference fluid and test fluid behaved as Newtonian fluids. Both fluids had the same value, as *μ_ref_* = *μ_test_* = 1 cP. The interface between both fluids was relocated by varying the flow rate ratio of the reference fluid to test fluid. As shown in Figure A2A (Appendix A), when the interface moved from center line (*α_x_* = 0.5) to each wall (i.e., *α_x_* = 0 or 1), the blood viscosity without the correction factor (i.e., *μ_n_*) showed a large deviation when compared with the viscosity of the test fluid (*μ_test_* = 1 cP). Considering that the viscosity of the test fluid should have a constant value of *μ_test_* = 1 cP with respect to the interface, the correction factor (C*_f_*) could be obtained by reciprocating *μ_n_* with respect to *α_x_* (i.e., *C_f_* = 1/*μ_n_*). By conducting a regression analysis, the variations of the correction factor with respect to the interface were obtained as $C_{f}=\;9.7212{\alpha_{x}}^{4}-19.442{\alpha_{x}}^{3}+\;15.687{\alpha_{x}}^{2}-5.9659\alpha_{x}+\;1.8992$ (*R*^2^ = 0.9968). As shown in Figure A2B (Appendix A), to validate *C_f_*, the viscosities of the test fluid were given as (a) *μ_test_* = 1 cP and (b) *μ_test_* = 4.08 cP. The flow rates of both fluids were the same, at 1 mL/h (i.e., *Q_ref_* = *Q_test_* = 1 mL/h). By applying the correction factor, the viscosities of the test fluids were determined as 1 cP and 4.14 cP, respectively. From the results, it was found that the parallel co-flowing method with the correction factor had the ability to measure the viscosity of a test fluid within 1.4% of a normalized difference. However, at *t* > *T_st_*, the continuous ESR inside the ACS caused unstable behaviors in blood flows. To quantify the ESR, two indices (i.e., *I_ESR,_ T_ESR_*) were suggested from <*I_B_*> and *T_st_*, as shown in Figure 5. Based on previous studies [45,50], one ESR index (*I_ESR_*) was suggested simply by integrating <*I_B_*> from *t* = *T_st_* to *t* = *T_end_* (i.e., $I_{ESR}=\int_{t=T_{st}}^{t=T_{end}}<I_{B}>dt$). Here, *T_end_* represented the end time of each experiment. Additionally, *T_ESR_* = *T_st_* − *T_i_*. *T_i_* indicated the initial time when the blood sample started to fill the blood channel.

From the preliminary demonstration, four factors (<*U_B_*>, <*U_R_*>, *α_B_*, and <*I_B_*>) could be effectively employed to obtain the blood viscosity and ESR when two ACSs were employed to deliver the blood sample and reference fluid into a microfluidic device.

### 2.4. Selection of Base Solution in Reference Fluid

**Figure A3:**
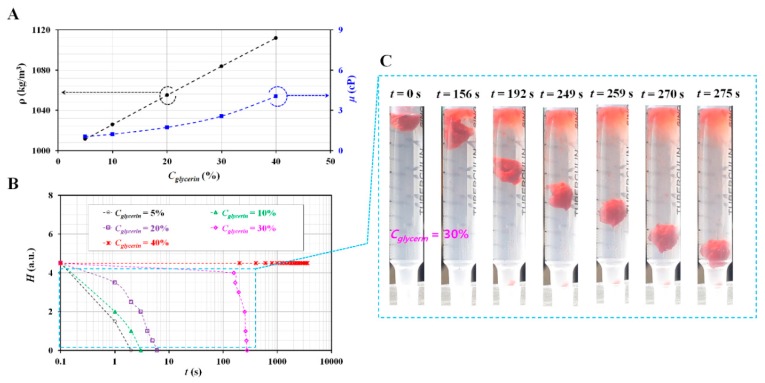
Selection of proper reference fluid by quantifying density (*ρ*) and sedimentation of RBCs droplet with respect to *C_glycerin_* = 5%, 10%, 20%, 30%, and 40%. (**A**) Variations of *ρ* and viscosity (*μ*) with respect to *C_glycerin_*. (**B**) Temporal variation of sedimentation height (*H*) with respect to *C_glycerin_*. (**C**) Snapshots for showing sedimentations of RBCs droplet with elapsed time (*t*) (*t* = 0, 156, 192, 249, 259, 270, and 275 s). Here, the syringe was filled with a 30% glycerin solution.

To visualize the velocity fields of the reference fluid, RBCs were added into the reference fluid as fluid tracers. Glycerin solution was suggested as a reference fluid to minimize the sedimentation of RBCs inside the ACS. According to a previous study [51], the density (*ρ*) and viscosity (*μ*) increased at higher concentrations of glycerin solution as shown in Figure A3A (Appendix A). To evaluate the sedimentation rate of the RBCs added into the reference fluid, a simple ESR tester was prepared by using a disposable syringe (~1 mL) as shown in Figure A3C (Appendix A). The disposable syringe was fitted vertically into a hole (outer diameter = 4 mm) of the PDMS block. The outlet of the hole was closed with 3M adhesive tape. The syringe was filled with a specific concentration of glycerin solution (~0.5 mL). A 50 μL RBCs droplet was dropped into a specific concentration of glycerin solution. To monitor the sedimentation rate of the RBCs droplet in the simple ESR tester, snapshots were captured at an interval of 1 s with a smartphone camera (Galaxy A5, Samsung, Korea). As shown in Figure A3B (Appendix A), temporal variations of sedimentation height (*H*) were obtained by varying the concentration of the glycerin solution (*C_glycerin_*) (*C_glycerin_* = 5%, 10%, 20%, 30%, and 40%). Figure A3C (Appendix A) showed sedimentation of the RBCs droplet in 30% glycerin solution over time (*t*) (*t* = 0, 156, 192, 249, 259, 270, and 275 s). From the results, the RBCs droplet in the 40% glycerin solution remained nearly identical at the upper position, even without sedimentation. Furthermore, considering that the densities of normal RBCs range from 1090 kg/m^3^ to 1106 kg/m^3^ [52], the reference fluid was selected as a 40% glycerin solution (*C_glycerin_* = 40%), because its density was greater than that of the RBCs.

### 2.5. Statistical Analysis

The statistical significance was evaluated by conducting statistical analyses with a commercial software package (Statistical Package for the Social Sciences (SPSS) Statistics version 24, IBM Corp., Armonk, NY, USA). Two ESR indices (*I_ESR_*, *T_ESR_*) and blood viscosity (<*μ_B_*>) obtained by the present method were compared with results reported in a previous study (i.e., blood viscosity: *μ_B_*, ESR index: *S_EAI_*). An analysis of variance (ANOVA) test was applied to verify significant differences between comparative results. A linear regression analysis was conducted to verify the correlations between two parameters. All experimental results were expressed as mean ± standard deviation. If the *p*-value was less than 0.05, the experimental results exhibited significant differences within a 95% confidence interval.

## 3. Results and Discussion

### 3.1. Contribution of RBCs Added into Reference Fluid to Viscosity and Velocity Fields

**Figure 2:**
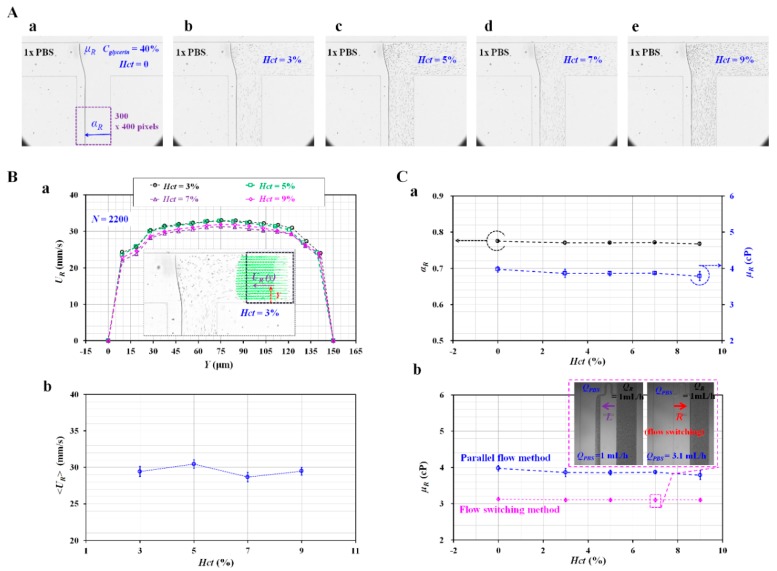
Contributions of RBCs added into the reference fluid to viscosity. 1× PBS was delivered to the blood channel at a constant flow rate of 1 mL/h with a syringe pump (SP). The hematocrit (*Hct*) of reference fluid was adjusted by adding normal RBCs into 40% glycerin solution (*Hct* = 0, 3%, 5%, 7%, and 9%). The reference fluid was delivered to the reference channel at a constant flow rate of 1 mL/h with an SP. (**A**) Microscopic images for obtaining interface (*α_R_*) in co-flowing channel with respect to *Hct* ((**a**) *Hct* = 0, (**b**) *Hct* = 3%, (**c**) *Hct* = 5%, (**d**) *Hct* = 7%, and (**e**) *Hct* = 9%). (**B**) Contributions of hematocrit in reference fluid to velocity fields (*U_R_*). (**a**) Variation of velocity fields (*U_R_*) across reference channel width with respect to *Hct*. The inset showed a microscopic image and a velocity profile of the reference fluid with *Hct* = 3%. (**b**) Variations of <*U_R_*> averaged over a region of interest (ROI) with respect to *Hct*. (**C**) Effect of *Hct* in reference to fluid on viscosity (*μ_R_*). (**a**) Variations of *α_R_* and *μ_R_* with respect to *Hct*. (**b**) Comparison between the proposed method (i.e., the parallel-flow method with correction factor) and previous method (i.e., flow-switching method) with respect to *Hct*.

To evaluate the effects of the RBCs added into the reference fluid on fluid viscosity, the viscosity of the reference fluid was measured by varying the volume of the RBCs added to the reference fluid (i.e., hematocrit [*Hct*]). In that regard, a 1× PBS was delivered to the blood channel (i.e., left-side channel) at a constant flow rate of *Q_PBS_* = 1 mL/h with a syringe pump (SP) (neMESYS, Centoni Gmbh, Germany). The hematocrit (*Hct*) of the reference fluid was adjusted to *Hct* = 3%, 5%, 7%, and 9% by adding normal RBCs into the 40% glycerin solution. The reference fluid was delivered to the reference channel (i.e., right-side channel) at a constant flow rate of *Q_glycerin_* = 1 mL/h with an SP. Figure 2A showed microscopic images for evaluating the interfacial location in the co-flowing channel with respect to *Hct* ((a) *Hct* = 0%, (b) *Hct* = 3%, (c) *Hct* = 5%, (d) *Hct* = 7%, and (e) *Hct* = 9%). To verify the contribution of the hematocrit in the reference fluid to the velocity fields (*U_R_*), the velocity fields of the reference fluid were obtained across the reference channel width with respect to *Hct*. As shown in Figure 2B-a, a variation of the velocity profile (*U_R_*) was obtained across the reference channel width with respect to *Hct*. The inset showed the microscopic image and velocity profile of the reference fluid with *Hct* = 3%. From the results, the velocity profile did not show a distinctive difference depending on the hematocrit. Figure 2B-b showed variations of the averaged velocity of the reference fluid (<*U_R_*>) with respect to *Hct*. The hematocrit in the reference fluid did not contribute to varying <*U_R_*> significantly. As shown in Figure 2C-a, the variations of the interface in the co-flowing channel (*α_R_*) and the viscosity (*μ_R_*) were obtained with respect to *Hct*. The interface and viscosity remained constant as *α_R_* = 0.771 ± 0.003 and *μ_R_* = 3.868 ± 0.068 cP for the reference fluid, with up to 9% hematocrit. From the results, it could be observed that providing up to a 9% hematocrit in the reference fluid did not significantly contribute to increasing the viscosity of the reference fluid. In addition, as shown in Figure A3A (Appendix A), an empirical formula [51] indicated that a 40% glycerin solution without any RBCs had a viscosity value of 4.07 cP. Based on the parallel co-flowing method with the correction factor [32], the viscosity of the reference fluid was measured consistently within a 5% difference when compared with the empirical formula. Furthermore, a previous flow-switching method [14] was employed to measure the viscosity of the reference fluid with respect to hematocrit. The inset of Figure 2C-b showed reversal flow-switching in the junction channel for the reference fluid with *Hct* = 7%. By increasing the flow rate of the 1× PBS (*Q_PBS_*) from *Q_PBS_* = 1 mL/h to *Q_PBS_* = 3.1 mL/h, the hydrodynamic balancing in both side channels caused to reverse flow direction from left direction to right direction (i.e., reversal flow-switching phenomena) [14]. In other words, the junction channel was filled with blood at *Q_PBS_* = 1 mL/h. However, it was filled with 1× PBS at *Q_PBS_* = 3.1 mL/h. Based on the viscosity formula suggested in the flow-switching method, the viscosity of the reference fluid was quantified as *μ_R_* = 3.1 ± 0.05 cP. As shown in Figure 2C-b, the viscosity obtained by both methods remained stable, with respect to hematocrit. Similar to the case in the parallel co-flowing method with the correction factor, the results of the flow-switching method indicated that the RBCs added into the reference fluid did not contribute to varying the viscosity within 9% hematocrit. The viscosity obtained by the flow-switching method was underestimated by approximately 20% when compared with that obtained by the parallel co-flowing method with the correction factor. From these results, in this study, the reference fluid was prepared by adding normal RBCs (*Hct* = 7%) into a 40% glycerin solution throughout all experiments.

### 3.2. Relationship between Flow Rate of Syringe Pump and Averaged Velocity Obtained by μPIV

To obtain blood viscosity, the flow rates of the blood sample and reference fluid should be measured from the averaged velocity obtained by conducting the µPIV technique. In other words, there was a need to obtain the relationship between the flow rate delivered by the SP (*Q_sp_*) and the averaged velocity obtained by conducting the µPIV technique (<*U*>).

**Figure 3:**
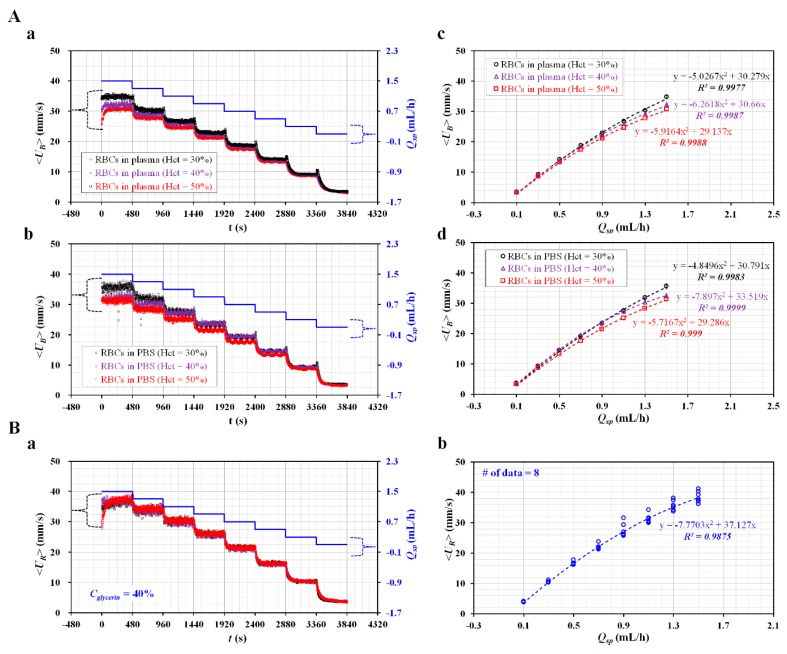
Calibration formula for the relationship between flow rate controlled by SP (*Q_sp_*) and averaged velocity obtained by µPIV technique (<*U*>). Hematocrit (*Hct*) of blood was adjusted to *Hct* = 30%, 40%, and 50% by adding normal RBCs into a base solution (1× PBS or plasma). With two syringe pumps, the flow rate of each fluid decreased stepwise from *Q_sp_* = 1.5 mL/h to *Q_sp_* = 0.1 mL/h at an interval of 0.2 mL/h. Each flow rate was maintained for 8 min. (**A**) Relationship between *Q_sp_* and <*U_B_*> with respect to hematocrit and base solution. (**a**) Temporal variations in <*U_B_*> and *Q_sp_* of blood sample (normal RBCs in plasma) with respect to *Hct*. (**b**) Temporal variations in <*U_B_*> and *Q_sp_* of blood sample (normal RBCs in 1× PBS) with respect to *Hct*. (**c**) Regression formula between <*U_B_*> and *Q_sp_* of blood sample (normal RBCs in plasma) with respect to *Hct*. (**d**) Regression formula between <*U_B_*> and *Q_sp_* of blood sample (normal RBCs in 1× PBS) with respect to *Hct*. (**B**) Calibration formula of relationship between *Q_sp_* and <*U_R_*> of reference fluid (40% glycerin solution with RBCs (*Hct* = 7%). (**a**) Temporal variations of *Q_sp_* and <*U_R_*>. (**b**) Regression formula between <*U_B_*> and *Q_sp_*.

The hematocrit of the blood sample was adjusted to *Hct* = 30%, 40%, and 50% by adding normal RBCs into the base solution (1× PBS, plasma). Using two SPs, the flow rate of each fluid decreased stepwise from *Q_sp_* = 1.5 mL/h to *Q_sp_* = 0.1 mL/h, at an interval of 0.2 mL/h. With respect to each flow rate, the SP had been operated for 8 min. The blood sample was prepared by adding normal RBCs into plasma. As shown in Figure 3A-a, temporal variations of the averaged velocity (<*U_B_*>) and the flow rate of the SP (*Q_sp_*) were obtained by varying the hematocrit. At a higher flow rate of *Q_sp_*, the hematocrit contributed to decreasing <*U_B_*>. At a lower flow rate of *Q_sp_*, <*U_B_*> remained constant, without contribution from the hematocrit. By changing the base solution from plasma to a 1× PBS, temporal variations of <*U_B_*> and *Q_sp_* were obtained with respect to *Hct*. As shown in Figure 3A-b, the variations of <*U_B_*>, with respect to hematocrit, were very similar to those of a blood sample composed of plasma. By averaging <*U_B_*> with respect to *Q_sp_*, <*U_B_*> was quantified as mean ± standard deviation with respect to *Q_sp_*. To determine the relationship between <*U_B_*> and *Q_sp_*, a scatter plot was used to plot <*U_B_*> on a vertical axis and *Q_sp_* on a horizontal axis. Figure 3A-c showed variations of <*U_B_*> with respect to *Q_sp_* and *Hct* in a blood sample composed of plasma. For example, <*U_B_*> was estimated as about 30 mm/s for *Q_sp_* =1.3 mL/h. Based on formula of flow rate (i.e., *Q_μP_* = <*U_B_*>A_c_, *A_c_* = w x h), flow rate obtained by μPIV was estimated as *Q_μPIV_* = 0.54 mL/h. When compared with *Q_sp_* =1.3 mL/h, the normalized difference between *Q_sp_* and *Q_μPIV_* was estimated as 59%. In this study, instead of the flow rate formula, the flow rate of the blood sample or reference fluid was estimated from the calibration formula obtained in advance. Thus, it was necessary to determine the relationship between velocity (<*U_B_*>) and *Q_sp_*. A regression analysis was conducted by assuming the regression formula as a quadratic model. Regression formulas between <*U_B_*> and *Q_sp_* with respect to *Hct* were obtained, as shown inside of Figure 3A-c. The regression formulas for each hematocrit were obtained as <*U_B_*> = −5.027 *Q_sp_*^2^ + 30.279 *Q_sp_* (*R*^2^ = 0.998) for *Hct* = 30%, <*U_B_*> = −6.262 *Q_sp_*^2^ + 30.660 *Q_sp_* (*R*^2^ = 0.999) for *Hct* = 40%, and <*U_B_*> = −5.916 *Q_sp_*^2^ + 29.137 *Q_sp_* (*R*^2^ = 0.999) for *Hct* = 50%. Figure 3A-d showed variations of <*U_B_*> with respect to the *Q_sp_* and *Hct* in a blood sample composed of 1× PBS. From the regression analysis, as shown inside Figure 3A-d, the regression formulas for each hematocrit were obtained as <*U_B_*> = −4.850 *Q_sp_*^2^ + 30.791 *Q_sp_* (*R*^2^ = 0.998) for *Hct* = 30%, <*U_B_*> = −7.897 *Q_sp_*^2^ + 33.519 *Q_sp_* (*R*^2^ = 1.000) for *Hct* = 40%, and <*U_B_*> = −5.717 *Q_sp_*^2^ + 29.286 *Q_sp_* (*R*^2^ = 0.999) for *Hct* = 50%. For the same hematocrit, the base solution (i.e., plasma or 1× PBS) did not contribute to varying the coefficients of the quadratic formula (i.e., normalized difference < 4% except *Hct* = 40%). However, for the same base solution, the coefficients of a quadratic model varied significantly with respect to hematocrit.

A regression formula between *Q_sp_* and <*U_R_*> for the reference fluid (i.e., 40% glycerin solution) with RBCs (*Hct* = 7%) was obtained by using a similar procedure to that used for the blood sample. Figure 3B-a showed the temporal variations of *Q_sp_* and <*U_R_*> for the reference fluid. <*U_R_*> was obtained as a mean ± standard deviation for a corresponding *Q_sp_*. When compared with the blood sample, <*U_R_*> increased substantially, owing to the lower value of the hematocrit. As shown in Figure 3B-b, variations of <*U_R_*> with respect to *Q_sp_* were represented by a scatter plot. From a regression analysis, the regression formula between <*U_R_*> and *Q_sp_* was obtained as <*U_R_*> = −7.770 *Q_sp_*^2^ + 37.127 *Q_sp_* (*R*^2^ = 0.9875).

From the results, the coefficients of the quadratic formula were varied significantly with respect to hematocrit. However, the base solution (i.e., 1× PBS, or plasma) did not contribute to changing the coefficients of the regression formula. Using regression formulae between *Q_sp_* and <*U_R_*> (or <*U_B_*>) obtained in advance, the <*U_R_*> or <*U_B_*> obtained by conducting the µPIV technique was converted into a flow rate (i.e., *Q_B_*, *Q_R_*, respectively).

### 3.3. Quantitative Comparison of Blood Viscosity with Respect to Fluid Delivery System (ACS, SP)

Since the <*U_R_*> and <*U_B_*> obtained from the µPIV technique were converted into flow rates (*Q_R_* and *Q_B_*) from regression formulae obtained in advance, the blood viscosity could be measured by monitoring the interface (*α_B_*) in the co-flowing channel, under fluid delivery with an ACS. The blood viscosity obtained by the ACS was quantitatively compared with one obtained by an SP. Blood samples (*Hct* = 30%, 40%, and 50%) were prepared by adding normal RBCs into the base solution (1× PBS, plasma).

**Figure 4:**
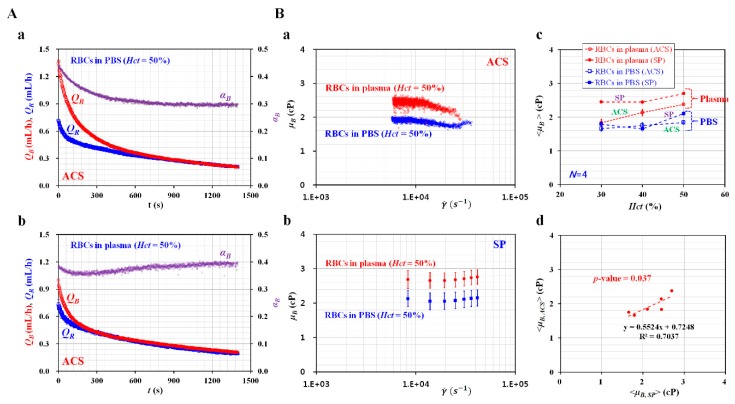
Quantitative comparison of blood viscosity for blood samples (normal RBCs in plasma and PBS, *Hct* = 50%) with respect to the fluid delivery system (ACS, SP). (**A**) Variations of flow rates (*Q_B_*, *Q_R_*) and interface (*α_B_*) with respect to the base solution (1× PBS, plasma). (**a**) Temporal variations of *Q_B_*, *Q_R_*, and *α_B_* for a blood sample (normal RBCs in 1× PBS, and *Hct* = 50%). (**b**) Temporal variations of *Q_B_*, *Q_R_*, and *α_B_* for a blood sample (normal RBCs in plasma, and *Hct* = 50%). (**B**) Variation of blood viscosity depending on the base solution, hematocrit, and fluid delivery system (ACS and SP). (**a**) Variations of blood viscosity (*μ_B_*) of blood samples with respect to the base solution (1× PBS, plasma) and shear rate under fluid delivery of ACS. (**b**) Variations of *μ_B_* with respect to the base solution (1× PBS, plasma) and shear rate under fluid delivery of two SPs. (**c**) Variations of <*μ_B_*> with respect to base solution (1× PBS, plasma), hematocrit (*Hct* = 30%, 40%, and 50%), and fluid delivery system (ACS, SP). <*μ_B_*> was quantified as <*μ_B_*> = mean ± standard deviation by conducting an arithmetic average of *μ_B_* obtained over shear rates. (**d**) Correlation between blood viscosity obtained under ACS (<*μ_B_*, *_ACS_*>) and blood viscosity obtained under SP (<*μ_B_*, *_SP_*>).

Figure 4A-a showed the temporal variations of *Q_R_*, *Q_B_*, and *α_B_* for the blood sample (normal RBCs in 1× PBS, *Hct* = 50%). In addition, Figure 4A-b depicted the temporal variations of *Q_R_*, *Q_B_*, and *α_B_* for the blood sample (normal RBCs in plasma, *Hct* = 50%). Using the blood viscosity formula, the blood viscosity was obtained by using the temporal variations of *Q_R_*, *Q_B_*, and *α_B._* Here, the viscosity of the reference fluid was given as *μ_R_* = 4.08 cP by using measurement results reported in previous studies [14,51]. For a rectangular channel (width = *w*, depth = *h*) with a lower aspect ratio [32], the formula of shear rate ($\dot{\gamma }$) was given as approximately $\dot{\gamma }=\frac{6Q_{B}}{wh^{2}}$. The corresponding shear rate of the blood viscosity obtained at a specific blood flow rate (*Q_B_*) was estimated by using the shear rate formula. A scatter plot was employed to plot *μ_B_* on a vertical axis, and $\dot{\gamma }$ on a horizontal axis. As shown in Figure 4B-a, variations of *μ_B_* were obtained with respect to the shear rate under fluid delivery with the two ACSs. Here, the blood sample (*Hct* = 50%) was prepared by adding normal RBCs into plasma or 1× PBS. The blood sample has behaved as a Newtonian fluid at sufficiently higher shear rates ($\dot{\gamma }>10^{3}\;s^{-1}$). From the experimental results, *μ_B_* remained constant with respect to the shear rate. By conducting an arithmetic average of *μ_B_* over specific shear rates, the blood viscosity was expressed as <*μ_B_*> = mean ± standard deviation. The viscosity of the blood sample composed of plasma (<*μ_B, plasma_*> = 2.381 ± 0.042 cP) was significantly higher than that of the blood sample composed of 1× PBS (<*μ_B, PBS_*> = 1.845 ± 0.0573 cP). To compare with the blood viscosity obtained under fluid delivery with two ACSs, the same blood samples were employed to measure the blood viscosity under fluid delivery with two SPs. Two fluids (blood sample, reference fluid) were delivered to each inlet of the microfluidic device, at the same flow rate (*Q_B_* = *Q_R_*).

As represented in Figure 3A-a and Figure 3A-b, the flow rate of the SP (*Q_sp_*) decreased stepwise from *Q_sp_* = 1.5 mL/h to *Q_sp_* = 0.1 mL/h at an interval of 0.2 mL/h. Each flow rate had been maintained for 8 min. As shown in Figure 4B-b, variations in *μ_B_* of the blood samples (normal RBCs in plasma and 1× PBS, *Hct* = 50%) were obtained with respect to the shear rate. The blood viscosity remained constant with respect to the shear rate. The viscosity of the blood sample composed of plasma (<*μ_B, plasma_*> = 2.728 ± 0.0918 cP) was higher than that of the blood sample composed of 1× PBS (<*μ_B, PBS_*> = 2.109 ± 0.0429 cP). When compared with the blood viscosity obtained by the ACS, blood viscosity obtained by the SP increased by approximately 12.5%. To determine the effects of hematocrit on blood viscosity, variations of blood viscosity were obtained by varying the hematocrit (*Hct* = 30%, 40%, and 50%), base solution (1× PBS, plasma), and fluid delivery system (ACS, SP). Figure 4B-c showed the variations of <*μ_B_*> with respect to *Hct*, base solution, and the fluid delivery system. Under fluid delivery with an ACS, <*μ_B_*> tended to increase with respect to *Hct*. Under fluid delivery with an SP, there was no significant difference between *Hct* = 30% and *Hct* = 40%. The blood viscosity increased at *Hct* = 50% when compared with *Hct* = 30% or 40%. To determine the correlation between the blood viscosity obtained by the ACS (<*μ_B, ACS_*>) and the blood viscosity obtained by the SP (<*μ_B, SP_*>), a scatterplot was used to plot <*μ_B, ACS_*> on a vertical axis, and <*μ_B, SP_*> on a horizontal axis, as shown in Figure 4B-d. According to a linear regression analysis, <*μ_B, ACS_*>was expressed as <*μ_B, ACS_*>*=* 0.5524 <*μ_B, SP_*> + 0.7248 (*R*^2^ = 0.7037, *p*-value = 0.037). Here, *p-value* = 0.037 indicated that a linear regression showed sufficient relationship between two viscosity values (i.e., <*μ_B_*_, ACS_> vs. <*μ*_B, SP_>). In addition, *R*^2^ was obtained as a high value of *R*^2^ = 0.7037. Although two SPs were effectively used to deliver two fluids during measurement of blood viscosity, the arrangement included challenges, such as a bulky size and a high cost. From the correlation between <*μ_B, ACS_*> and <*μ_B, SP_*>, it was found that the ACS can be effectively employed to deliver two fluids in the measurement of blood viscosity. Thus, the blood viscosity can be measured consistently under fluid delivery with two ACSs.

### 3.4. Quantitative Measurement of ESR with Respect to base Solution and Hematocrit

The ESR of the blood sample was evaluated by quantifying the microscopic image intensity of the blood sample (<*I_B_*>) flowing in the blood channel. Two ESR indices (*t_ESR_*, *I_ESR_*) were suggested by quantifying the temporal variations of <*I_B_*>. The blood samples (*Hct* = 30%, 40%, and 50%) were prepared by adding normal RBCs into a base solution (1× PBS, plasma).

**Figure 5:**
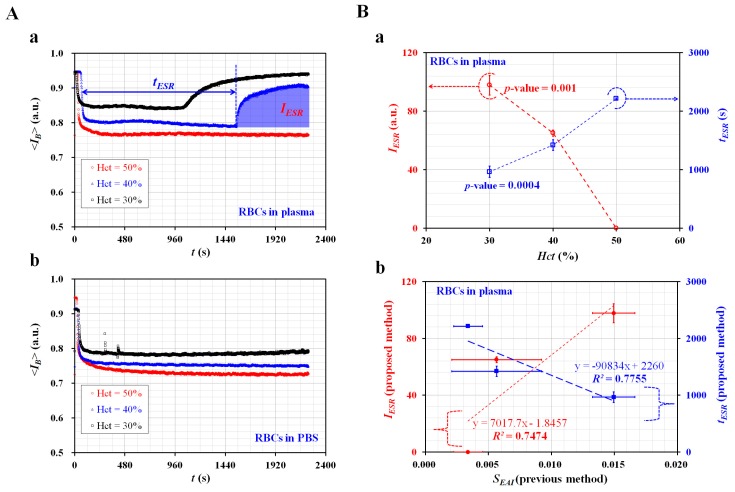
Evaluation of ESR for blood samples with respect to base solutions (1× PBS, and plasma) and hematocrit (*Hct* = 30%, 40%, and 50%). (**A**) Variations of <*I_B_*> with respect to base solution and hematocrit. (**a**) Temporal variations of <*I_B_*> of blood sample (normal RBCs in plasma) with respect to *Hct*. (**b**) Temporal variations of <*I_B_*> of blood sample (normal RBCs in 1× PBS) with respect to *Hct*. (**B**) Variations of two ESR indices (*t_ESR_*, *I_ESR_*) for blood sample (normal RBCs in plasma) with respect to *Hct*. (**a**) Variations of *t_ESR_* and *I_ESR_* with respect to *Hct*. (**b**) Correlation between proposed ESR indices (*t_ESR_*, *I_ESR_*) and previous ESR index (*S_EAI_*).

As shown in Figure 5A-a, variations of <*I_B_*> for the blood sample (normal RBCs in plasma) were obtained with respect to *Hct*. <*I_B_*> tended to decrease at higher values of *Hct*. In addition, *T_st_* tended to be shorter at lower values of the hematocrit. To exclude the contribution of plasma protein to the ESR, the plasma was replaced with the 1× PBS. As shown in Figure 5A-b, temporal variations of <*I_B_*> for the blood sample (normal RBCs in 1× PBS) were obtained by varying *Hct*. <*I_B_*> tended to decrease at higher values of *Hct*. With a certain elapse of time, <*I_B_*> remained constant. There was no existence of separation time within 2000 s (i.e., *T_st_* > 2000 s). The results indicated that the 1× PBS did not sufficiently contribute to enhancing ESR when compared with plasma.

To quantify the ESR of the blood sample (normal RBCs in plasma) from <*I_B_*> as shown in Figure 5A-a, two ESR indices (*t_ESR_*, *I_ESR_*) were obtained with respect to the hematocrit. Figure 5B-a showed variations of *t_ESR_* and *I_ESR_* with respect to *Hct*. According to the results, *t_ESR_* tended to increase significantly with respect to hematocrit (*p*-value = 0.0004). *I_ESR_* tended to decrease substantially with respect to hematocrit (*p*-value = 0.001). Under blood delivery with the ACS, the RBCs tended to fall down continuously inside the ACS, which was installed horizontally. Owing to the continuous ESR inside the ACS, the populations of RBCs delivered to the blood channel decreased over time. Thus, <*I_B_*> increased gradually over time, as shown in Figure 5A-a. However, when the plasma was replaced with the 1× PBS, the blood sample did not exhibit an ESR inside the ACS. For this reason, after a certain period of time, <*I_B_*> remained constant over time, as shown in Figure 5A-b. To quantitatively compare with results reported in a previous study, two indices (*t_ESR_*, *I_ESR_*) and *S_EAI_* (previous ESR index) [45] were plotted on a vertical axis and horizontal axis, respectively. *S_EAI_* exhibited larger scatters than *t_ESR_* or *I_ESR_*. From the regression analysis, the linear regression exhibited higher values of *R*^2^ = 0.7474~0.7755. The results indicated that the two ESR indices exhibited consistent variations with respect to hematocrit when compared with *S_EAI_*. Thus, the two ESR indices (*t_ESR_*, *I_ESR_*) can be effectively used to evaluate the variation of ESR with respect to hematocrit.

### 3.5. Variations of Blood Viscosity and ESR for Blood Samples Composed of Specific Dextran Solutions

**Figure 6:**
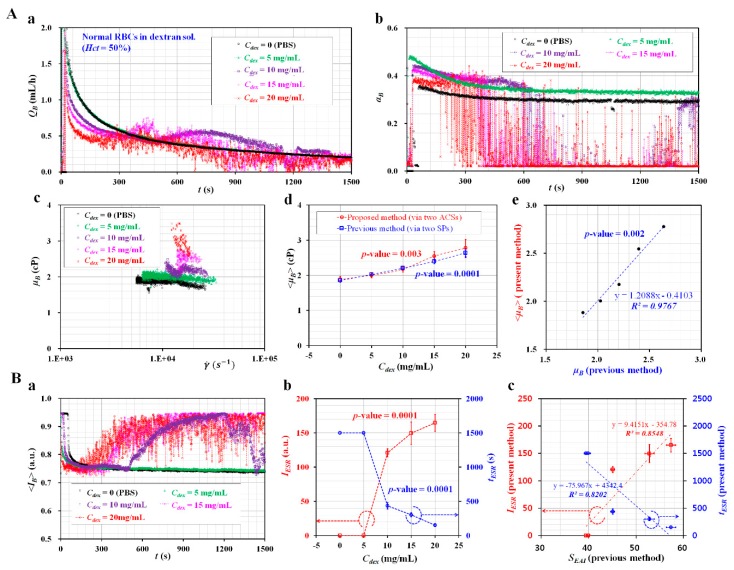
Measurement of blood viscosity and ESR for blood samples composed of specific concentrations of dextran solution. Blood samples (*Hct* = 50%) were prepared by adding normal RBCs into various concentration of dextran solution (*C_dex_*) (i.e., *C_dex_* = 0, 5, 10, 15, and 20 mg/mL). *C_dex_* = 0 meant 1× PBS as control. (**A**) Variations of blood viscosity with respect to *C_dex_*. (**a**) Temporal variations of *Q_B_* with respect to *C_dex_*. (**b**) Temporal variations of *α_B_* with respect to *C_dex_*. (**c**) Variations of *μ_B_* with respect to shear rate and *C_dex_*. (**d**) Variations of <*μ_B_*> with respect to the *C_dex_* and fluid delivery system (ACS, SP). (**e**) Correlation between blood viscosity obtained by the proposed method and blood viscosity obtained by the previous method. (**B**) Variations of ESR with respect to *C_dex_*. (**a**) Temporal variations of <*I_B_*> with respect to *C_dex_*. (**b**) Variations of two ESR indices (*t_ESR_*, *I_ESR_*) with respect to *C_dex_*. (**c**) Quantitative comparison between the proposed method (*t_ESR_*, *I_ESR_*) and previous method (*S_EAI_*).

A specific dextran solution as a base solution was prepared to enhance the ESR of the blood sample. To exclude the contributions of hematocrit to ESR, the hematocrit of the blood sample was adjusted to *Hct* = 50%. The blood samples were prepared by adding normal RBCs into specific concentrations of dextran solution (i.e., *C_dex_* = 0, 5, 10, 15, and 20 mg/mL). *C_dex_* = 0 meant 1× PBS as control. As shown in Figure 6A-a, temporal variations of *Q_B_* were obtained with respect to *C_dex_*. From the results, the blood sample composed of dextran solution (*C_dex_* <= 5 mg/mL) exhibited stable variations of *Q_B_* over time. However, above *C_dex_* >= 10 mg/mL, the separation time (*T_st_*) tended to reduce at higher concentrations of the dextran solution. Figure 6A-b showed temporal variations of *α_B_* with respect to *C_dex_*. Similar to the case with *Q_B_*, *α_B_* varied unstably above *C_dex_* >= 10 mg/mL. *T_st_* tended to be shorter at higher concentrations of the dextran solution. By using stable variations of *Q_B_* and *α_B_* obtained at *t* < *T_st_*, variations of *μ_B_* were obtained with respect to the shear rate. As shown in Figure 6A-c, blood viscosities were obtained at sufficiently higher shear rates ($\dot{\gamma }>10^{3}\;s^{-1}$). They remained constant with respect to the shear rate. Additionally, the blood viscosity tended to increase at higher concentrations of the dextran solution. By averaging the *μ_B_* values obtained at shear rates, the blood viscosity was expressed as <*μ_B_*> = mean ± standard deviation. Figure 6A-d showed variations of <*μ_B_*> with respect to *C_dex_* and the fluid delivery system (ACS, SP). When compared with the results reported in a previous study [45], the present results exhibited sufficiently consistent variations of <*μ_B_*> with respect to *C_dex_*. In addition, there was no significant difference between the ACS and SP. As shown in Figure 6A-e, to determine the correlation between <*μ_B_*> obtained by the proposed method (two ACSs) and *μ_B_* obtained by the previous method (two SPs) [45], a scatter plot was used to plot <*μ_B_*> (proposed method) on a vertical axis and *μ_B_* (previous method) on a horizontal axis. According to linear regression analysis, the high value of *R*^2^ = 0.9767 indicated that the proposed method could give comparable results when compared with the previous method. Thus, ACSs could be effectively employed to deliver fluid samples. After measuring the blood viscosity with respect to *C_dex_*, the contributions of the dextran solution to the ESR were evaluated by quantifying the image intensity of the blood sample flowing in the blood channel (<*I_B_*>). As shown in Figure 6B-a, temporal variations of <*I_B_*> were obtained with respect to *C_dex_*. As a result, *T_st_* was reduced at higher concentrations of the dextran solution. Using <*I_B_*> with respect to *C_dex_*, two ESR indices (*t_ESR_*, *I_ESR_*) were obtained with respect to *C_dex_*. As shown in Figure 6B-b, the ESR indices remained constant up to *C_dex_* = 5 mg/mL. Above *C_dex_* >= 10 mg/mL, *t_ESR_* tended to decrease substantially with respect to *C_dex_* (*p*-value = 0.0001). *I_ESR_* increased largely at higher concentrations of dextran solution (*p*-value = 0.0001). To compare with the results reported in a previous study [45], a scatterplot was used to plot *t_ESR_* and *I_ESR_* (i.e., proposed method) on a vertical axis, and *S_EAI_* (i.e., previous method: periodic on-off control with an SP) on a horizontal axis. As shown in Figure 6B-c, a linear regression analysis was conducted to determine the correlation between the proposed method and the previous method. The higher value of *R*^2^ = 0.8202–0.8548 indicated that the two ESR indices (*t_ESR_*, *I_ESR_*) gave comparable results when compared with the previous method. Thus, the ESR indices can be effectively used to quantify the ESRs of blood samples.

### 3.6. Variations of Blood Viscosity and ESR for Blood Samples Composed of Hardened RBCs

As the last demonstration, the proposed method was applied to evaluate the contribution of hardened RBCs to the ESR. As shown in Figure 5A-a, a blood sample (*Hct* = 50%) composed of plasma did not contribute to variations in the ESR. To stimulate the ESR of a blood sample with a high value of *Hct* = 50%, the plasma as a base solution was replaced with a specific concentration of dextran solution (i.e., *C_dex_* = 15 mg/mL). Hardened RBCs were prepared by sufficiently exposing normal RBCs to specific concentrations of GA solution (*C_GA_*) (i.e., *C_GA_* = 0, 5, 10, and 15 μL/mL). *C_GA_* = 0 indicated normal RBCs as control. The blood samples (*Hct* = 50%) were then prepared by adding hardened RBCs into the specific dextran solutions.

**Figure 7:**
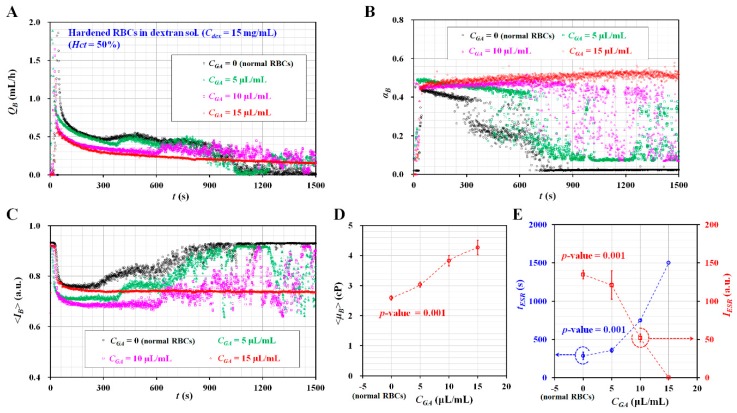
Measurement of blood viscosity and ESR and blood viscosity for blood samples composed of hardened RBCs with glutaraldehyde (GA) solution. To prepare hardened RBCs from normal RBCs, normal RBCs were sufficiently exposed to specific concentrations of GA solution (*C_GA_*) (*C_GA_* = 0, 5, 10, and 15 μL/mL). *C_GA_* = 0 meant normal RBCs as control. Blood sample (*Hct* = 50%) was prepared by adding hardened RBCs into the dextran solution (*C_dex_* = 15 mg/mL). (**A**) Temporal variations of *Q_B_* with respect to *C_GA_*. (**B**) Temporal variations of *α_B_* with respect to *C_GA_*. (**C**) Temporal variations of <*I_B_*> with respect to *C_GA_*. (**D**) Variations of <*μ_B_*> with respect to *C_GA_*. (**E**) Variations of two ESR indices (*t_ESR_*, *I_ESR_*) with respect to *C_GA_*.

Figure 7A showed the temporal variations of *Q_B_* with respect to *C_GA_*. *T_st_* tended to increase at higher concentrations of the GA solution. At *C_GA_* = 15 μL/mL, *Q_B_* tended to decrease stably over time. Figure 7B showed the temporal variations of *α_B_* with respect to *C_GA_*. The variations of *α_B_* were very similar to those of *Q_B_*. At *C_GA_* = 15 μL/mL, *α_B_* remained constant after a certain period of time. Figure 7C showed the temporal variations of <*I_B_*> with respect to *C_GA_*. Except for at *C_GA_* = 15 μL/mL, <*I_B_*> tended to increase stably over time. Additionally, *T_st_* tended to increase at higher concentrations of the GA solution. By measuring three factors (*Q_B_*, *α_B_*, and <*I_B_*>) simultaneously, the hardened blood sample composed of hardened RBCs fixed with *C_GA_* = 15 μL/mL did not exhibit an ESR inside the ACS. Thus, there were no significant variations of *Q_B_* and *α_B_*. As shown in Figure 7D, variations of <*μ_B_*> were obtained with respect to *C_GA_*. <*μ_B_*> tended to increase considerably with respect to *C_GA_*. As the GA solution contributed to stiffening the RBCs’ membranes, it was reasonable that the blood viscosity increased at higher concentrations of the GA solution. Figure 7E showed variations of the two ESR indices (*t_ESR_*, *I_ESR_*) with respect to *C_GA_*. *t_ESR_* tended to increase with respect to *C_GA_*. *I_ESR_* tended to decrease with respect to *C_GA_*.

From the experimental results, it was found that the GA solution caused an increase in blood viscosity. Furthermore, the proposed method had the ability to consistently measure blood viscosity and ESR, under simultaneously fluid delivery from two ACSs.

## 4. Conclusions

In this study, a simple method of measuring blood viscosity and ESR was demonstrated by quantifying averaged velocities of a blood sample and reference fluid, where the blood sample and reference fluid were delivered to a microfluidic device with two ACSs. According to the experimental results, a 40% glycerin solution with RBCs (*Hct* = 7%) was selected as the reference fluid to obtain velocity fields and avoid sedimentation of RBCs in the ACS. Using a calibration formulae between the flow rate of an SP (*Q_sp_*) and the averaged velocity obtained by the µPIV technique (<*U_B_*>) in advance, <*U_B_*> or <*U_R_*> was converted into *Q_B_* or *Q_R_*, respectively. As a demonstration, the proposed method was employed to evaluate the contributions of the hematocrit (*Hct* = 30%, 40%, and 50%), base solution (1× PBS, plasma, dextran solution), and hardened RBCs to the blood viscosity and ESR, respectively. The results of the proposed method were comparable with those reported in previous studies that used two SPs. From the experimental results, it could be concluded that the proposed method had the ability to consistently measure blood viscosity and ESR under simultaneous fluid delivery with two ACSs. However, image acquisition for quantifying blood flows in microfluidic channels was demonstrated from the optical microscope and a high-speed camera. To resolve the issue, the proposed method should be improved substantially by adopting a portable image acquisition system in the near future.

## Figures and Tables

**Figure A4:**
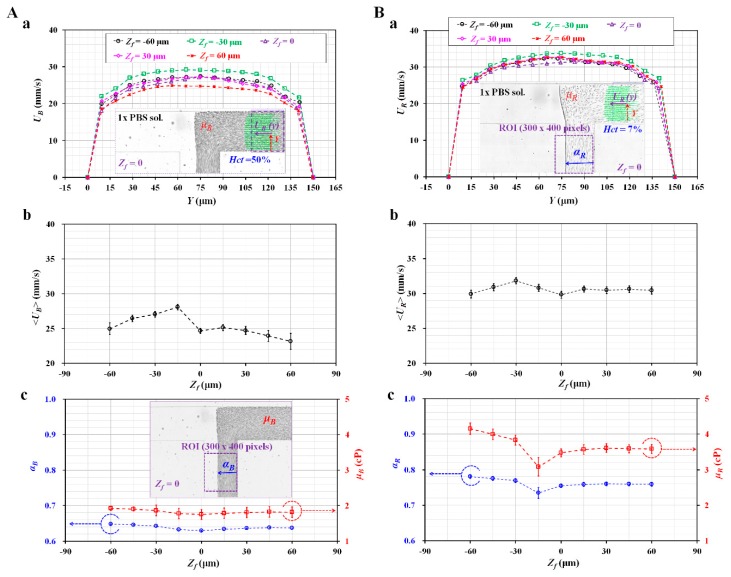
Effect of the relocation of object plane (*Z_f_*) on velocity, interface, and viscosity with respect to the blood sample and reference fluid. A blood sample (*Hct* = 50%) was prepared by adding normal RBCs into a 1× PBS. A reference fluid with RBCs (*Hct* = 7%) was prepared by adding normal RBCs into a 40% glycerin solution. Both fluids were delivered to inlets at a constant flow rate of 1 mL/h with two SPs. (**A**) Effects of the relocation of object plane on velocity, interface, and viscosity for the blood sample. (**a**) Variations of *U_B_* across reference channel width with respect to *Z_f_* = −60, −30, 0, 30, and 60 μm. The inset depicted the velocity profile of the blood sample flowing in the reference channel at *Z_f_* = 0. (**b**) Variations of <*U_B_*> with respect to *Z_f_*. (**c**) Variations of *α_B_* and *μ_B_* with respect to *Z_f_*. The inset showed interfacial location (*α_B_*) at *Z_f_* = 0. (**B**) Effect of the relocation of object plane on velocity, interface, and viscosity for reference fluid with RBCs (*Hct* = 7%). (**a**) Variations of *U_R_* across reference channel width with respect to *Z_f_* = -−60, −30, 0, 30, and 60 μm. The inset represented *U_R_* and *α_R_* at *Z_f_* = 0. (**b**) Variations of <*U_R_*> with respect to *Z_f_*. (**c**) Variations of *α_R_* and *μ_R_* with respect to *Z_f_*.

## Appendix A

### Appendix A.4. Blood Sample Preparation

According to the protocol approved by the Ethics Committee of Chosun University Hospital (CUH) (CHOSUN 2018-05-11), all experimental procedures were conducted after confirming that the procedures involved were appropriate and humane.

Human concentrated RBCs and fresh frozen plasma (FFP) were purchased from the Gwangju-Chonnam blood bank (Gwangju, Korea). The concentrated RBCs and FFP were kept at 4 °C and −20 °C, respectively. The concentrated RBCs were preserved in an anticoagulant solution (i.e., citrate phosphate dextrose adenine [CPDA]). To remove the CPDA from the concentrated RBCs, washing procedures were performed twice. The concentrated RBCs (~20 mL) were mixed with phosphate-buffered saline (PBS) (1×, pH 7.4, Gibco, Life Technologies, New York, NY, USA) (~20 mL) in a 40 mL tube. After inserting the tube in a centrifuge (Allegra X-30R benchtop, Beckman Coulter, Brea, CA, USA), the centrifuge was set to 4000 rpm and was operated for 10 min. Owing to differences in density, the blood sample was distinctively separated into two layers (i.e., upper layer: liquid, and lower layer: RBCs) in the tube. Normal RBCs were collected after removing the liquid positioned in the upper layer. To completely remove the anticoagulant solution, a washing procedure was repeated twice. The FFP was thawed under a room temperature of 25 °C. For removing debris existing in the FFP, pure plasma was collected by passing the FFP through a syringe filter (mesh size = 5 μm, Minisart, Sartorius, and Germany). Finally, the normal RBCs and plasma were stored at 4 °C in a refrigerator before the experiment. Various blood samples were prepared by adding normal or hardened RBCs into a specific base solution. Except for experiments to determine the contributions of hematocrit to blood viscosity or ESR, the hematocrit of the blood sample was fixed at *Hct* = 50% for consistent measurement.

First, to evaluate the contributions of hematocrit to blood viscosity and ESR, the hematocrit of the blood sample was adjusted to *Hct* = 30%, 40%, and 50% by adding normal RBCs into base solution (i.e., 1× PBS, and plasma). Second, to enhance the ESR in ACS, five different concentrations of dextran solution (i.e., *C_dex_* = 0, 5, 10, 15, and 20 mg/mL) were prepared by adding dextran powder (*Leuconostoc* spp., MW = 450–650 kDa, Sigma-Aldrich, USA) into 1× PBS. Then, blood samples (*Hct* = 50%) were prepared by adding normal RBCs into specific concentrations of the dextran solution. Third, four concentrations of glutaraldehyde (GA) solution (i.e., *C_GA_* = 0, 5, 10, and 15 µL/mL) were diluted by mixing the GA solution (Grade II, 25% in H_2_O, Sigma-Aldrich, USA) with 1× PBS. Homogeneous hardened RBCs were prepared by exposing normal RBCs to each concentration of the GA solution for 10 min. To enhance ESR in blood sample (*Hct* = 50%) significantly, plasma was replaced with a specific concentration of dextran solution (*C_dex_* = 15 mg/mL). A hardened blood sample (*Hct* = 50%) was then prepared by adding homogeneous hardened RBCs into the specific dextran solution.

### Appendix A.5. Variation of Velocity, Interface, and Viscosity with Respect to Relocation of Object Plane

Variations of velocity, interface, and viscosity were evaluated by moving an object plane (*Z_f_*) in the depth direction. The object plane (*Z_f_*) relocated from *Z_f_* = −60 μm to *Z_f_* = 60 μm at intervals of 15 μm. *Z_f_* = 0 represented that the microscopic images were captured at the focus plane (i.e., the best conditions for focus). *Z_f_* > 0 meant that the microfluidic device moved vertically and that the microscopic images were captured at an out-of-focus plane. *Z_f_* < 0 meant that the microfluidic device moved in a gravitational direction and that the microscopic images were captured at the out-of-focus plane. The blood sample (*Hct* = 50%) was prepared by adding normal RBCs in a 1× PBS. The reference fluid with RBCs (*Hct* = 7%) was prepared by adding normal RBCs into a 40% glycerin solution. Using two SPs, the reference fluid or blood sample was delivered to the reference channel (i.e., right-side channel). Simultaneously, the 1× PBS was delivered to the blood channel (i.e., left-side channel). The flow rate of each fluid remained at a constant flow rate of 1 mL/h.

First, with respect to the blood sample, the contribution of the object plane to velocity fields and viscosity was evaluated with respect to the relocation of the object plane. As shown in Figure A4-(A-a), variations of the velocity profile of the blood sample (*U_B_*) across the reference channel width were obtained with respect to *Z_f_* = −60, −30, 0, 30, and 60 μm. Using data sets of *n* = 180, each velocity was averaged and expressed as a mean ± standard deviation. The inset of Figure A4-(A-a) depicted the velocity profile of blood flow (*U_B_*) estimated from sequential microscopic images captured at the focal plane (*Z_f_* = 0). When *Z_f_* increased from *Z_f_* = 0 to an out-of-focus plane, *U_B_* tended to decrease. In other words, the velocity fields varied depending on the object plane.

Based on an analytical formula for a depth of correlation (DOC) suggested in a previous study [53], the DOC was estimated as 33.9 µm. Since the DOC was sufficiently higher than the channel depth of 20 µm (i.e., DOC > depth), all RBCs in the microfluidic channel could contribute to calculating the velocity fields uniformly. According to the estimated DOC, the µPIV technique measured averaged velocity fields in the depth direction at the focal plane (*Z_f_* = 0). The averaged velocities of the blood sample and reference fluid (<*U_R_*>, and <*U_B_*>) were calculated as an arithmetic average over the specific ROI.

Figure A4-(A-b) showed variations of <*U_B_*> with respect to *Z_f_*. When *Z_f_* increased from the focal plane (*Z_f_* = 0) to an out-of-focus plane, <*U_B_*> tended to gradually decrease. The averaged blood velocity (<*U_B_*>) remained constant from *Z_f_* = 0 to *Z_f_* = 30 μm. From the results, <*U_B_*> remained constant until the microfluidic device moved vertically with regard to DOC. In that regard, when *Z_f_* decreased from *Z_f_* = -15 μm, <*U_B_*> tended to gradually decrease.

Figure A4-(A-c) showed variations of *α_B_* and *μ_B_* with respect to *Z_f_*. The inset of Figure A4 (A-c) showed the interfacial location of blood flow (*α_B_*) in a microscopic image captured at the focal plane (*Z_f_* = 0). The blood viscosity (*μ_B_*) was obtained by inserting *α_B_* into the viscosity formula as reported in previous studies [32,33]. As *α_B_* and *μ_B_* remained constant with respect to *Z_f_*, the relocation of the object plane did not contribute to varying the blood viscosity.

Second, with respect to the reference fluid with RBCs (*Hct* = 7%), the effects of the object plane on velocity and viscosity were evaluated by quantifying variations of the velocity fields and viscosity when the object plane moved vertically. As shown in Figure A4-(B-a), variations of velocity profile (*U_R_*) were obtained with respect to *Z_f_* = -60, -30, 0, 30, and 60 μm. The inset of Figure A4-(B-a) represented *U_R_* at the focal plane (*Z_f_* = 0). Except for *Z_f_* = -30 μm, the relocation of the object plane did not contribute to varying significant variations of <*U_R_*>. Figure A4-(B-b) showed variations of <*U_R_*> with respect to *Z_f_*. When *Z_f_* relocated vertically from the focal plane (*Z_f_* = 0), <*U_R_*> remained constant with respect to *Z_f_*. From the results, <*U_R_*> remained constant, even though the object plane relocated vertically outside the DOC. However, when *Z_f_* relocated in a gravitational direction from the focal plane (*Z_f_* = 0), <*U_R_*> fluctuated along the object plane. As shown in Figure A4-(B-c), variations of *α_R_* and *μ_R_* were obtained with respect to *Z_f_*. Similar to <*U_R_*>, *α_R_* and *μ_R_* remained constant when *Z_f_* relocated in the vertical depth direction. However, *α_R_* and *μ_R_* exhibited large fluctuations and increased when relocating *Z_f_* in a gravitational direction.

From these experimental results, it was found that the averaged velocities of the blood sample and reference fluid (<*U_B_*>, and <*U_R_*>) remained constant throughout the relocation of the object plane outside the DOC. In other words, when a microfluidic device moved up to the DOC vertically, the averaged velocity and interface remained constant. However, when a microfluidic device moved in the gravitational direction, it contributed to fluctuations of velocity, interface, and viscosity. For consistent measurement of velocity fields and the interfacial location, a microfluidic device was positioned at *Z_f_* = 0–30 μm. In other words, a microscopic image was then captured at the focus plane or at a slightly out-of-focus plane.
